# Hydranencephaly: cerebral spinal fluid instead of cerebral mantles

**DOI:** 10.1186/s13052-014-0079-1

**Published:** 2014-10-18

**Authors:** Piero Pavone, Andrea D Praticò, Giovanna Vitaliti, Martino Ruggieri, Renata Rizzo, Enrico Parano, Lorenzo Pavone, Giuseppe Pero, Raffaele Falsaperla

**Affiliations:** Unit of Pediatrics and Pediatric Emergency, University Hospital “Policlinico-Vittorio Emanuele”, Catania, Italy; Department of Formative Processes, University of Catania, Catania, Italy; Chair of Child Neuropsychiatry, University of Catania, Catania, Italy; Institute of Neurological Sciences, National Research Council, Catania, Italy; Neuroradiologic Unit, Department of Radiology, University of Catania, Catania, Italy

**Keywords:** Hydranencephaly, Holoprosencephaly, Congenital anomaly, Brain malformation, Severe hydrocephalus

## Abstract

The authors report a wide and updated revision of hydranencephaly, including a literature review, and present the case of a patient affected by this condition, still alive at 36 months.

Hydranencephaly is an isolated and with a severe prognosis abnormality, affecting the cerebral mantle. In this condition, the cerebral hemispheres are completely or almost completely absent and are replaced by a membranous sac filled with cerebrospinal fluid. Midbrain is usually not involved. Hydranencephaly is a relatively rare cerebral disorder. Differential diagnosis is mainly relevant when considering severe hydrocephalus, poroencephalic cyst and alobar holoprosencephaly. Ethical questions related to the correct criteria for the surgical treatment are also discussed.

## Introduction

Hydranencephaly (HE) is a rare, mostly isolated abnormality, which is reported to affect about 1 out 5000 continuing pregnancies [1,2]; an accurate incidence is difficult to determine, considering how similar this condition is to others and the limited diagnostic techniques that have been available in the past. It is one of the most severe forms of bilateral cerebral cortical anomaly. In this condition, the cerebral hemispheres are completely or almost completely missing. In their place, there is a membranous sac filled with cerebrospinal fluid, glial tissue and ependyma. There is, however, preservation of the skull. There is variable and partial involvement of the inferior frontal, temporal and occipital lobes. The midbrain, cerebellum, thalami, basal ganglia, and choroid plexus are usually not involved [3-5]. Hydranencephaly may affect only one hemisphere (hemihydrancencephaly- HHE) [6,7], which is even rarer than the bilateral form and, in some cases, allows for a better prognosis. In spite of the recent advances in diagnostic tools to recognize HE, many doubts persist on the etiopathogenetic aspects, time of onset, and it is often misdiagnosed due to the similarities of HE to disorders involving the cerebral mantle (Table 1).

**Table 1:** **Differential diagnosis of hydranencephaly with hydrocephalus**, **holoprosencephaly**, **porencephaly**

	**Hydranencephaly**	**Hydrocephalus**	**Holoprosencephaly**	**Porencephaly**
**Head circumference**	Normal or slightly smaller	Larger	Normal	Normal
**Midline malformations**	Absent	Absent	Present	Absent
**Brainstem anomalies**	Absent	Absent	Present	Absent
**Intact cortical rim**	Absent	Present	Present	Present
**Dilated third ventricle**	Absent	Present only in obstructive forms	Absent	Absent
**Angiographic investigation**	Bilateral internal carotid artery occlusion (not always)	Normal	Normal	Involvement of middle cerebral artery resulting in localized areas of cortical destruction
**Facial malformations**	Absent	Absent	Present	Absent
**Surgical treatment**	Doubt	Useful	Not advisable	Not advisable

The aim of our review is to report on this condition, utilizing data from the literature, and also presenting our experience with a patient affected by the bilateral form of HE, who is still alive at the age of 36 months. The references for this review were obtained from the Medline database (PubMed.gov; U.S. National Library of Medicine- National Institutes of Health using the search term “hydranencephaly”).

### Historical background

Cruveilhier [8] reported two cases of patients with symptoms resembling HE in his book “Anatomie Pathologique du Corps Humain” between the years 1829–1835, and has been accredited by Bettinger [9] to be the first to describe a case of HE. Indeed, Cruveilhier [8] reported on two different types of anencephaly, which he defined as hydrocephalic anencephaly and as anencephaly presenting with a complete absence of the skull. The term hydranencephaly was presumably first introduced by Spielmeyer [10] in 1905, in a paper entitled “Ein hydranencephales Zwillingspaar”, in which the author reported this anomaly in a pair of female dichorionic twins. An autopsy performed on these patients showed the cerebral remnants to have haemorrhagic lesions and blood pigments and signs of nerve tissue involvement. The blood vessels showed thin walls and cavernous spaces and the haemorrhagic lesions were referred to as vascular anomalies [10]. According to Marburg and Casamajor [11], venous stasis and thrombosis, especially involving the deep cerebral veins, were supposed to be evidence for which HE was pathogenetically responsible. Picaza et al. [12] suggested the name hydranencephalodysplasia, as this term was more inclusive of any condition affecting the cerebral mantle associated with hydrocephalus. Crome and Sylvester [13] suggested the term hydranencephaly to highlight the clinical difference with anencephaly, in which the cerebral vault is involved. However, the term “hydranencephaly” has been considered more appropriate and most of the experts prefer to use it, as it is more descriptive. Since its identification, many case-reports have been published, describing clinical and pathologic aspects and associations of HE with infectious and teratogenic sources. McAbee et al. [14], in a paper published in 2000, described two patients with HE with prolonged survival at 66 and 24 months, respectively. They also reported data from the literature, collecting examples of 15 patients, including 4 who were also described by Sutton et al. [15]. Recently, Cecchetto et al. [16] in an extensive and current review of cases collected between 2000 and 2012, report the experience of 4 patients, two of whom experienced prolonged survival: a 32-year-old male and a 14-year-old female. Among the lists of patients reported in the literature, the series described by Adeloye [17] is the largest, involving 15 patients with HE.

### Epidemiology

Hydranencephaly is a rare neurological condition, but it has been more frequently reported in the literature than the unilateral form, for which only nine cases have been published [6,7]. This may be due to the different pathogenetic events inducing the two different forms, and perhaps, also to missed HE diagnosis in favor of similar disorders. In fact, as many as 1% of infants thought to have hydrocephalus by clinical examination were later found to have hydranencephaly [18] and vice versa. Cecchetto et al. [16], in their review, reported information from 37 different publications on this topic and collected examples of 76 patients from the literature.

### Etiopathogenesis

The etiopathogenesis of HE is still unknown, however most researchers support the hypothesis that the brain damage in HE is related to early internal carotid artery involvement, as demonstrated by a) angiographic and autoptic observation, in which internal carotid artery anomalies, both aplastic and hypoplastic, are reported and b) by the anatomic distribution of the anomaly in HE, which follows the internal carotid artery supply [14,16,18]. Thus, HE is categorized as a member of a group of circulatory developmental encephalopathies. Two main hypotheses have been advanced to explain the severe brain impairment. One is the destructive theory, for which the hydranencephaly occurs after the brain and ventricles have partially or fully formed, and are then destroyed in utero, due to an encephaloclastic process. The anomalous event would occur after neural migration and before synaptogenesis, probably during the second trimester. It is during this phase that the fetal cerebral hemispheres present a relevant transformation, with wide proliferation, which will generate the diencephalon, mesencephalon and the entire brainstem [7]. The second hypothesis refers to a dysontogenetic process with an early disruption of organogenesis [18]. These hypotheses cannot be confirmed, since there are no examples of HE in which the brain previously appeared normal at an MRI or ultrasound examination and was then destroyed [16]. Molecular dysfunctions, as with those reported in other cases of cerebral anomalies, may also be postulated, as was reported in a patient with porencephaly, resembling HE, in which a COL4A1 mutation was found [19] or PI3K-Akt3-mTOR mutations for neurodevelopmental anomaly and specifically for megalencephaly [20-22]. Cytogenetic anomalies (triploidy) were observed in two of the four patients reported by Cecchetto et al. [16]. While the hypothesis of vascular anomaly seems the most probable, this cannot be confirmed in all the cases reported in the literature where, at the autoptic investigations, the internal carotid artery was not impaired. According to Cecchetto et al. [16] the normality of internal carotid arteries in HE does not exclude the possible pathogenetic role in the impairment of the HE brain, since the internal carotid arteries might canalize again after having caused the severe brain damage. However, without under evaluating the various pathogenetic hypotheses, the occlusion of cerebral arteries above the supraclinoid level seems to be the most probable hypothesis [16].

The time it takes before evidence of brain anomalies can be detected with HE is still debated. Most of the case-reports have been diagnosed between the thirteenth and the twenty-sixth week of pregnancy, during the second trimester of gestation, the time in which the hemispheres and the falx cerebri have been already formed. There are, however, reports of HE patients diagnosed very early in the first trimester, before the twelfth gestational week [23]. This raises the possibility that the pathologic event causing HE may occur very early in the neurogenic phase.

While the pathogenesis of hydranencephaly is thought to be mainly linked to a vascular accident, the etiologic events that may interfere with normal vascular development are various and frequently reported. Intrauterine infections, particularly toxoplasmosis [24] and viral infections (enterovirus, adenovirus, parvovirus, cytomegalic, herpes simplex, Epstein-Barr, and respiratory syncytial viruses) have been implicated in a number of cases [25-28]. Toxic exposures, such as smoking and cocaine abuse, [29-32] estrogens [33,34] and sodium valproate [35] have also been reported. HE has been associated with young maternal age [36] and has been described in association with rare syndromes. HE has also been seen in monochorionic twin pregnancies, in which the death of one twin provoked a vascular exchange to the living twin through the placental circulation and may have caused hydranencephaly in the surviving fetus [37,38]. Deficiency of factor XIII (fibrin stabilizing factor) [39] and intracerebral haemorrhage [40] have also been suggested as causal events. All these reported causes could act directly or indirectly on the internal carotid arteries or in other cerebral districts, causing the profound brain alterations. According to these results, HE must be considered to be not a malformation, but rather a disruption, secondary to several pathological events, which cause ischemia in utero in the area of the carotid artery. A predisposing molecular anomaly has still not been demonstrated but cannot be excluded.

### Neuronal-pathologic features

In HE, the loss of cortex is massive but seldom complete. In the review by Cecchetto et al. [16], the remaining cerebral areas included: 6 cases of remaining fronto-occipital lobe, 2 frontal, 4 occipito-temporal, 1 fronto-temporal, 6 occipital, 2 fronto-occipito-parietal and 1 fronto-temporo-occipital. Remnant parts typically correspond to where in the vast, but somewhat variable, forebrain territory the anterior cerebral circulatory matrix connects. There is no uniformity of data in the autoptic investigations in the reported literature. The average case shows variable remnants of cortex supplied by the posterior circulation, notably inferomedial occipital, but also basal portions of the temporal cortex, and midline cortical tissue along the falx, extending into medial frontal cortex may be spared. Most of the cortical area is missing and in its place is a thin-walled, fluid-filled cyst. Brainstem, cerebellum, thalamus and striatum are usually preserved with a normal histological structure, also demonstrated by the neurophysiological investigations (evoked potentials) [41-43]. The cerebrospinal fluid circulation is open, but may be athresic. The thin membrane replacing the hemispheric wall is composed of immature nervous tissue elements: glial cells and connective tissue admixture [9,16,44,45] with a notable absence of neurons. The cyst tends to continuously enlarge, due to the increased fluid pressure. In a microscopic examination, such tissue may be found to be gliotic or to exhibit other structural anomalies indicating loss of function [3,4,45]. Olfactory and optic nerves are often spared.

### Clinical manifestations

During pregnancy, the mother typically feels normal fetal movements. At birth the child with HE usually shows a normal head circumference. With most of the cerebral cortex absent, the fetal head would be expected to be small. Although this may sometimes occur, the head is more often normal or increased in size because the choroid plexus within the lateral ventricles continue to produce cerebral spinal fluid that is not adequately absorbed. In some cases this can cause increased pressure, which may expand the head and lead to rupture of the falx cerebri [18].

Most affected children die before birth. Those who survive do not initially show evident neurological or clinical signs; archaic reflexes, leg and arms movements are usually normally present at birth, as are sucking and swallowing reflexes. However, more subtle signs, such as feeble crying, difficulty with feeding, hypotonia or wide anterior fontanelle may be present. The signs become rapidly more pronounced after a few days, presenting with severe hypotonia, irritability and seizures. The convulsive episodes are prevalently of the myoclonic type and may derive from the rim of the preserved cortex, but the attacks may also originate directly from the brainstem. In the children who survive, visual impairment, spastic diplegia and cognitive delay are usually reported.

**Figure 1:**
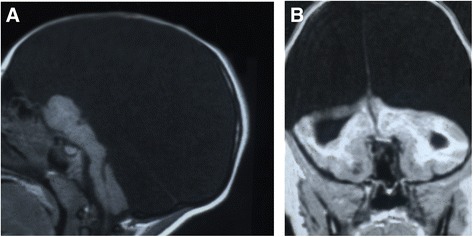
**AB: Sagittal (A) and choronal (B) projections of cerebral T1 weighted MRI performed on a patient affected by hydranencephaly.** The cerebral hemispheres are completely missing, replaced by a membranous sac filled with cerebrospinal fluid. Skull, brainstem and basal ganglia are preserved.

In January 2013, a 3 year-old female was referred to the Unit of Pediatrics and Pediatric Emergency, at the University Hospital “Vittorio Emanuele-Policlinic”, Catania, Italy for macrocephaly and hydrocephalus. She was the second child of unrelated Libian parents; the mother was aged 28, the father 34. The pregnancy was uneventful and ultrasound analysis was not performed. The parents confirmed that from the first months of life, the girl suffered from severe hypotonia, developmental delay and drug-resistant epileptic attacks. Upon physical examination, the girl’s weight and height were under the third percentile but her head circumference was in the 97^th^ percentile. The neurologic examination displayed a severe neurodevelopmental delay, spastic palsy with brisk patellar tendon reflexes. The child was unable to walk without support. Chest and heart examination were normal as well the abdominal organs. No other malformative anomalies were present. Routine laboratory analysis showed normal results including full blood count, coagulation tests, blood lactate and pyruvate, glucose and ketones, copper, ceruloplasmin, plasma and urine aminoacids. Antibodies against measles, and TORCH agents were negative. Karyotype was 46, XX. Video-EEG performed while awake and during sleep showed a diffuse flattened activity and slow waves and some sporadic paroxysmal activity. The brainstem auditory evoked response (BAER) to the right ear stimulation revealed wave peaks I through VII with normal thresholds, morphology and latency. Brain MRI showed the classic picture of HE (Figure 1 A, B). Due to MRI investigation results and the progressive head enlargement, the parents, following the advice of the neurosurgeon, decided to submit the child to ventriculo-peritoneal shunt. No real clinical improvement was observed six months later.

According to the literature, children affected by hydranencephaly show mental delay, spasticity and seizures. Hearing is usually preserved with rare cases of bilateral sensorineural hearing impairment, while vision tends to be compromised. Hypotelorism, bilateral optic nerve hypoplasia, diffused chorioretinal atrophy, pigment clumping and dysplastic retina have been reported [46].

Based on reported cases, family history is negative for HE and similar disorders. Both males and females are affected. Electroencephalography (EEG) in patients with HE shows absence of electric activity in all leads with a diffuse flattened tracing, or low amplitude, slow wave with poor modulation and no background. In other cases, the EEG may present with discharges of diffuse spikes and waves. Loss of cortical activity with preservation of brainstem function is confirmed by BAER, auditory middle latency responses, cortical auditory evoked responses, strobe electroretinograms, strobe-flash visual evoked responses and median and tibial nerve somatosensory evoked responses [41]. In some cases a total absence of visual evoked potential can be detected [42].

Brain ultrasonography shows absence of cerebral hemispheres and intact cerebellar vermis. The brain-MRI shows an almost total parenchymal absence, replaced by cerebrospinal fluid, frequently containing remnants of the occipital area and orbitofrontal regions. The falx is usually present, with the preservation of cerebellar hemispheres and the cerebral bulbus. Magnetic resonance phase contrast flow images may demonstrate internal carotid artery hypoplasticity or atresia [47].

### Diagnosis

**Figure 2:**
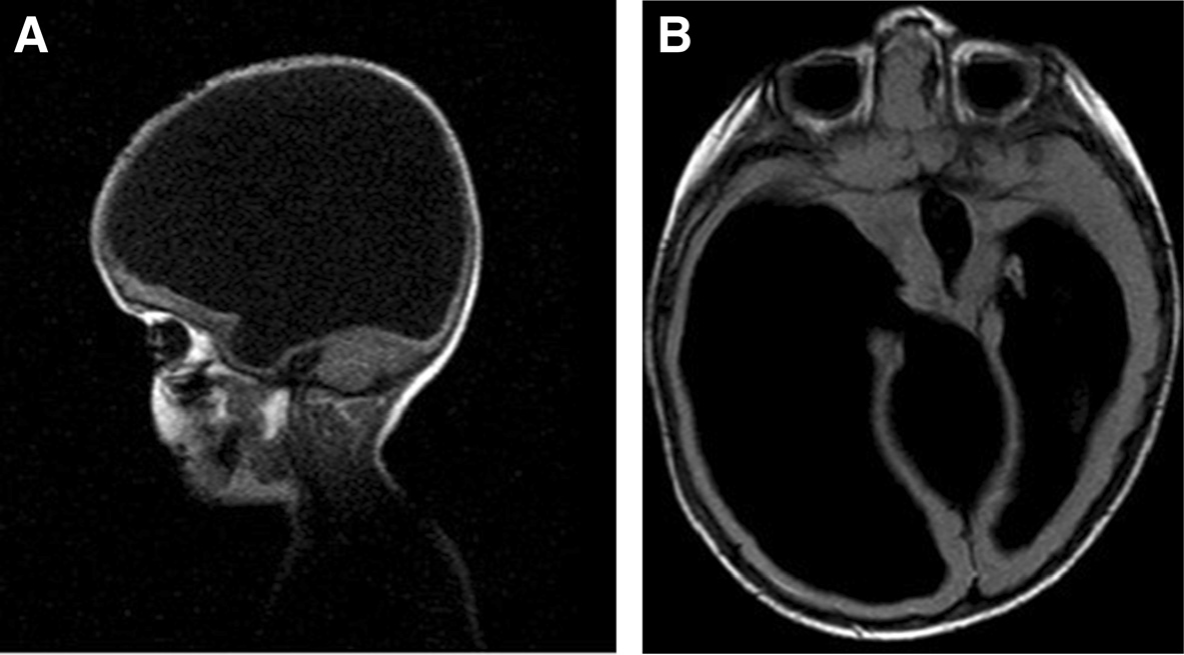
**AB: Sagittal (A) and axial (B) projections of Cerebral T1-weighted MRI performed on a patient affected by severe congenital hydrocephalus.** The images show the absence of cerebral hemisphere with only a small portion of the right frontal lobe and a rime of the left frontal and parietal lobes. Third ventricle is present and enlarged.

Diagnosis of HE can be determined in utero by ultrasonography. Ultrasound, CT scan and MRI are commonly used to make the diagnosis. Ultrasound examination could be useful in the prenatal diagnosis of HE and shows at 21 and 23 gestational weeks the absence of cerebral hemispheres, replaced by homogeneous echogenic material filling the supratentorial space, and preservation of the thalami, brain stem, and cerebellum [48]. After delivery, the golden standard examinations are CT and MRI scan, which permit to differentiate more precisely HE from holoprosencephaly or severe congenital hydrocephalus (Figure 1 and 2). Furthermore, transillumination could be useful in the diagnosis of HE, in absence of other possible neuroradiological examination. Based on neurological signs and physical examination of the head, brain MRI remains the best diagnostic test. EEG may be useful to confirm the presence and type of seizures, as it relates to their treatment. Ocular and auditory testing is also advisable. AngioMRI may be useful to find anomalies of the vascular cerebral structures.

### Differential diagnosis

Differential diagnosis is mainly relevant when considering severe hydrocephalus, poroencephalic cyst and alobar holoprosencephaly (Table 1). Diagnosis with severe congenital hydrocephalus is particularly difficult. Hydrocephalus is more frequent than HE, with an incidence of 1 in 1000 births. This condition has several recognized causes, including teratogens, embryopathic events and malformative syndromes as with Arnold-Chiari type II malformation, secondary to spina bifida. In the last condition, hydrocephalus is the most widespread and severe form, and is secondary to acqueductal stenosis. The distinction of hydrocephalus from the HE is based on the presence in the former of an intact cortical rim, and when the hydrocephalus is caused by aqueductal stenosis, the dilated third ventricle may be an important element in the differential diagnosis. Moreover, the head circumference in patients with HE is most often normal at birth or even slightly smaller. Rare is an HE baby born with head circumference rivalling the size of an infant with untreated hydrocephalus. The angiographic investigation showing bilateral internal carotid artery occlusion is relevant to determine a diagnosis of HE [18]. Differential diagnosis between severe congenital hydrocephalus and hydrancencephaly is particularly difficult but of notable importance in terms of prognosis and treatment response. Sutton and associates [15] followed 10 neonates with severe cerebral impairment; five infants had HE and five had hydrocephaly. The patients were followed up for 4–23 months with serial computed tomography, electroencephalograms and developmental evaluations. The five infants with HE, diagnosed after 1 month of age, despite aggressive surgical management and shunt placement, showed no neurological or radiological improvement. The five infants with severe hydrocephalus, who had been treated with a shunt placement, showed relevant clinical improvement.

Poroencephalic cysts are usually located in the middle cerebral artery territory with ischemic infarcts that subsequently result in localized areas of cortical destruction. Differential diagnosis of HE is however difficult. Frequently, the preservation of the frontal and parieto-occipital cortex, as frequently happens with poroencephalic cysts, may be a major factor in the diagnosis. Holoprosencephaly is a congenital cerebral anomaly, resulting from absent or incomplete diverticulation of the forebrain. The alobar form is the most severe. Unlike with HE, in the alobar form of holoprosencephaly, there is partial fusion of the thalami and the falx cerebri is missing. Moreover, the head in holoprosenphalic children is small and facial malformation is present.

### HE associated syndromes and malformations

HE, which manifests as an isolated anomaly, nevertheless has sometimes been reported in association with other conditions. Taori et al. [46] reported a case of HE associated with cerebellar involvement and bilateral microphthalmia and colobomas and Watts et al. [49] reported chorioretinal dysplasia and intracranic calcifications. Kelly et al. [50] reported on cerebellar hypoplasia, a posterior calcified mass related to the occlusion of the posterior cerebral arteries, and Mittelbronn et al. [51] described HE in association with polymicrogyria and feto-fetal transfusion in triplets and Segawa et al. [52] in association with holoprosencephaly in patient with partial 14q abnormality. HE has been reported to be associated with dysfunctions such as renal dysplasia [53,54], penoscrotal transposition with deletion (13) (q22) [55], PEHO-like dysmorphism [47], hypoplastic thumb [56], Poland syndrome [57] and lethal multiple pterygium syndrome [58]. HE is one anomaly among many of an autosomal-recessive syndrome characterized by proliferative vasculopathy and hydranencephaly-hydrocephaly known as Fowler syndrome [58-62]. Mutations in FLVCR2 has recently been reported in association with this syndrome [63].

### Prognosis

Unlike to the unilateral form, the prognosis of HE is usually quite poor. Affected patients mostly die in utero. In the survivors, death usually occurs in the first year of life. Developmental delay, drug-resistant seizures, spastic diplegia, severe growth failure and respiratory infections are features which burden the life of these patients and are frequent causes of their death. However patients with survival of 20 [64], 22 [65] and 32 [16] years have been reported in the literature. The survival of the patient is related to the integrity of the brainstem, which regulates vital aspects, such as temperature, blood pressure, and cardiorespiratory function [14].

### Treatment

For the child with HE who survives, there is debate as to whether or not to perform any surgical treatment, considering the severe brain impairment. The surgical treatment consists of ventriculoperitoneal shunt, which drains the fluid, reducing the cerebral tension and the progressive increase of the cerebral volume. Recently, an optional treatment for selected patients has been proposed, consisting in choroid plexus coagulation (CPC). CPC plus endoscopic third ventriculostomy may increase the shunt independent rate, preventing the late complications related to shunt device [66,67]. According to the opinion of neurosurgeons repeated and even complex surgery such as choroid plexectomy may be useful [68]. In any case in our opinion, surgical treatment should at least be proposed to the family of patients with severe intracranial hypertension giving them the possibility to choose this option.

For surviving children, physiokinesitherapy, drugs for epileptic seizures and intervention in nutritional care are mandatory, as is the treatment of respiratory compromise.

## Conclusions

HE is a rare disease with in-utero death as the most typical result and a very poor life expectancy. However, the importance of such a diagnosis cannot be overlooked. Diagnosis of HE does introduce some ethical problems, however. First of all, the importance of a timely diagnosis of HE cannot be ignored, as it may help the family process emotional issues. Another important ethical question concerns the correct criteria for performing an abortion, considering that some children affected by less severe forms of HE may survive long term, even with severe cognitive and physical impairment and also that most of the surviving patients live in a vegetative state [69]. The third ethical question is the appropriate treatment for this disease. Should surgical intervention be advised when it offers only a small hope of success or should children be left without surgical treatment? Our patient recently underwent a surgical treatment of ventriculoperitoneal shunt but she did not reach sufficient improvement to justify the procedure.

It is obvious to the authors that the answer to these ethical questions, at least with current treatment options, is highly individual. The answer to these questions must be left to the parents, on whom the weight of this condition falls, until guidelines from ethical committees will be produced, with a univocal consensus in the medical care of these patients.
